# Metformin Decouples Phospholipid Metabolism in Breast Cancer Cells

**DOI:** 10.1371/journal.pone.0151179

**Published:** 2016-03-09

**Authors:** Tim A. D. Smith, Su M. Phyu

**Affiliations:** School of Medicine, Medical Sciences and Nutrition, University of Aberdeen, Foresterhill, Aberdeen, United Kingdom; Mayo Clinic College of Medicine, UNITED STATES

## Abstract

**Introduction:**

The antidiabetic drug metformin, currently undergoing trials for cancer treatment, modulates lipid and glucose metabolism both crucial in phospholipid synthesis. Here the effect of treatment of breast tumour cells with metformin on phosphatidylcholine (PtdCho) metabolism which plays a key role in membrane synthesis and intracellular signalling has been examined.

**Methods:**

MDA-MB-468, BT474 and SKBr_3_ breast cancer cell lines were treated with metformin and [^3^H-methyl]choline and [^14^C(U)]glucose incorporation and lipid accumulation determined in the presence and absence of lipase inhibitors. Activities of choline kinase (CK), CTP:phosphocholine cytidylyl transferase (CCT) and PtdCho-phospholipase C (PLC) were also measured. [^3^H] Radiolabelled metabolites were determined using thin layer chromatography.

**Results:**

Metformin-treated cells exhibited decreased formation of [^3^H]phosphocholine but increased accumulation of [^3^H]choline by PtdCho. CK and PLC activities were decreased and CCT activity increased by metformin-treatment. [^14^C] incorporation into fatty acids was decreased and into glycerol was increased in breast cancer cells treated with metformin incubated with [^14^C(U)]glucose.

**Conclusion:**

This is the first study to show that treatment of breast cancer cells with metformin induces profound changes in phospholipid metabolism.

## Introduction

Membrane phospholipids are key cell components with both a primary structural role, forming the basis of cell membranes, and a regulatory role providing pools of intermediates for intracellular signal transduction. The most abundant structural phospholipid in eukaryote cells is phosphatidylcholine (PtdCho) [1] whilst phosphatidylinositol (PtdIns) is a more minor membrane component but generates inositol 2,4,5 triphosphate for signal propagation downstream of many growth factor receptors including the tyrosine kinase human epithelial receptor family (HER) [2]. Ptdins and PtdCho are also important sources of the secondary messenger diacylglycerol [3] which is generated from the respective phospholipid by the action of phospholipid-specific phospholipase C (PLC).

Aberrant PtdCho metabolism is a characteristic of many cancers [4] due to changes in the activity of degradative enzymes including phospholipase C [5] and anabolic enzymes especially choline kinase [6,7]. Both choline kinase [6,7] and PtdCho-PLC [8] are essential for tumour progression and have been identified as potential cancer treatment targets[8,9].

Cancer cells have a high demand for fatty acids required for the synthesis of phospholipids for both new membrane synthesis and signalling. In contrast to normal cells, which generally utilise dietary fatty acids, many cancer cells exhibit a lipogenic phenotype involving increased activity of lipid metabolising enzymes, including fatty acid synthase (FAS) [10,11], in part induced by increased activation of Akt/mTor pathway [12]. Whilst the high fluxes of other pathways in tumour cells generates metabolites such as tricarboxylic acid [13] providing abundant sources of acetyl CoA for conversion to fatty acids. Fatty acid synthase (FAS) catalyses the synthesis of the long chain fatty acid from acetyl CoA and the resulting palmitic acid is then utilised in the production of cell phospholipids [14].

Metformin (1,1-dimethylbiguanide) is used in the treatment of type 2 diabetes (T2DM) as it lowers blood glucose levels, sensitises target cells to insulin [15] and decreases gluconeogenesis by the liver [16]. Metformin has been shown to improve the survival of cancer patients [17] whilst cancer risk in diabetic patients, which is increased compared with non-diabetic patients, has been shown to be decreased by treatment with metformin [18,19].

Metformin has consistently been shown to activate AMPK [20,21] which is believed to be triggered through inhibition of cytochrome 1 and consequent reduction in intracellular ATP concentration [20]. Other pathways including Akt, which regulates glucose metabolism [22] and lipid metabolism [23], have been shown to be modulated in the breast cancer cell line MDA-MB-231 by treatment with metformin but this appears to be cell-type dependent [21]. Inhibition of energy metabolism by treatment of prostate cancer cells with metformin has recently been shown to inhibit lipogenesis [24]. Other studies have demonstrated that metformin directly interferes with fatty acid synthesis in breast cancer cells by decreasing FAS activity [25]. The ability of metformin to inhibit cancer cell growth has been attributed in part to its inhibition of lipogenesis via activation of AMPK [26].

As metformin can modulate both glucose and fatty acid metabolism, which are key to the formation of the phospholipid precursor diacylglycerol, we have examined the effect of metformin on the rate of accumulation of PtdCho in breast cancer cells and the activities of Key enzymes involved in the formation (CK and CCT) and breakdown of PtdCho (PtdCho-PLC).

## Materials and Methods

### Materials

All chemicals were obtained from Sigma-Aldrich (Poole UK) unless otherwise stated. [^3^H-methyl]Choline chloride (60-90Ci/mmol, 1mCi/ml) was obtained from American Radiolabeled Chemicals Inc. (USA) and D-[^14^C(U)]Glucose (9.25–13.3GBq)/mmol) from Perkin Elmer (Beaconsfield UK). The phospholipase C inhibitor D609 [27],the triglyceride lipase inhibitor Atglistatin [28] and the acetyl CoA carboxylase α inhibitor TOFA [29] were obtained from Cambridge Biosciences (UK) and used at concentrations previously determined. Tissue culture media was obtained from Invitrogen (Paisley UK). Phosphate-buffered saline (PBS) was obtained from Fisher Scientific in 10X concentrated solution and diluted 10 fold with distilled water.

### Tissue culture

The breast cancer cell lines BT474, MDA-MB-468 and SKBr_3_ were obtained from the American Tissue Culture Collection (LGC Standards, Teddington UK) and maintained in Dulbecco’s Modified Eagles medium supplemented with penicillin (10,000units/100ml)/Streptomycin (10,000μg/100ml) and 10% foetal bovine serum in a CO_2_ incubator with humidified CO_2_:air (5%:95%) at 37°C.

### [^3^H-methy]Choline cellular incorporation

Cells were grown until confluent in 75cm^2^ tissue culture flasks (Nunclon, Thermo Scientific UK) and seeded (1x10^6^ cells per flask) into 25cm^2^ flasks. After 24h cells the media was replaced with either fresh medium or fresh medium with metformin (4mM) [21]. The flasks were then incubated for 72h unless otherwise stated.

[^3^H-methy]lcholine chloride uptake and phosphorylation: Media was replaced with 0.5ml of media containing [^3^H-methyl]choline chloride (185KBq/ml) and incubated at 37°C for 15min then washed rapidly 4x with ice cold phosphate buffered saline (PBS). The cells were then detached with 0.3ml of a solution of 0.05% trypsin: EDTA (Gibco UK) and neutralised with 0.3ml of medium. The cells were centrifuged at 1000g for 5min at 4°C and the media removed and retained. The cells were fractionated into lipid and aqueous phases by resuspension in 0.25ml of methanol (MeOH): CHCl_3_ (2:1) and placing on ice for 1h after which 0.125ml of CHCl_3_ and 0.125ml of Tris buffer (10mM) were added. The phases were then separated by centrifuging at 10,000g for 5min. The aqueous (upper phase) was then collected and the lipid (lower phase) placed in a scintillation vial (Perkin Elmer UK) with 3ml of scintillation fluid (Ultima Gold, Perkin Elmer UK) mixed and the activity counted on a TRI-CARB 2100TR liquid scintillation counter (Perkin-Elmer UK). Phosphorylated and non-phosphorylated [^3^H-methyl] Choline in the aqueous phase and the retained media were then determined as described below.

### Determination of Phosphorylated [^3^H-methyl] Choline by precipitation

To determine the amount of phosphorylated [^3^H-methyl]choline in the aqueous phase and medium the samples were made up to 1ml with water and 200ul of sample added to 3ml of scintillation fluid in a scintillation vial and counted. A further 200ul was mixed with 200ul of ZnSO_4_ (5% w/v) and 200ul of 0.3M Ba(OH)_2_. The mixture was then repeatedly mixed on a vortex mixer for 5min then centrifuged at 10,000g for 2min to pellet the precipitated phosphates. The radioactivity in a 200ul sample of the supernatant (containing non-phosphorylated [^3^H-methyl] Choline) was determined and the difference between the two samples, after allowing for dilution, was the amount of phosphorylated radioactivity [30].

### Determination of rate of [^3^H-methyl]choline incorporation into lipid

Cells were seeded into 25cm^2^ flasks, treated with metformin (4mM) for 72h then pulsed with [^3^H-methyl]choline as described for ‘[^3^H-methy]lcholine chloride uptake and phosphorylation’. After washing with ice-cold PBS the cells were incubated for 0 and 1h with fresh 0.5ml of non-radioactive medium (chase) after which the media was collected and the cells washed 2x with 0.2ml PBS and the washes pooled with the medium. Radioactivity was counted in the pooled media/ washes. The cells were trypsinised and radioactivity in the cells was determined after fractionation into lipid and aqueous phases as described above.

### Determination of [^14^C(U)]-glucose incorporation into lipids and glycerol

Cells were set up and treated as described for ‘[3H-methy]Choline cellular incorporation’ except they were incubated with media containing [^14^C(U)]glucose (185KBq/ml) for 2h then washed 5x with ice-cold PBS. The cells were detached by treatment with trypsin and after addition of medium, transferred to microfuge tubes and centrifuged at 400g for 5min. They were then washed 2x with ice-cold PBS and the washes and media pooled and radioactivity counted. The cells were then fractionated into lipid and aqueous fractions as described above ([^3^H-methy]Choline cellular incorporation). The radioactivity in a 50ul sample of the lipid fraction was counted and the solvent from the remainder evaporated. The lipids were then subjected to saponification by addition of KOH (10M) in water: ethanol (1:10) and heating to 70°C for 20min [31]. The fatty acids and glycerol were then separated into lipid and aqueous fractions [32] and radioactivity counted in the aqueous phase (glycerol) and the lipid phase.

### Thin layer chromatography (TLC)

Composition of lipid-phase: Lipid phase extracts were subject to TLC on aluminium backed silica-gel plates (Merck, Germany) using a mobile phase of chloroform/methanol/acetone/acetic acid/water (40:14:15:12:7 v/v) (modification of [33]. The plates were cut into 7mm pieces placed in scintillation vials. The silica gel was loosened by treatment with 0.1ml of NaOH (1M) and neutralised with HCl. After addition of scintillation fluid the bare aluminium pieces were removed and the samples counted. Standards consisting of pure phosphatidylcholine and lyso-phosphatidylcholine were run and visualised using iodine vapour. The two lipids appeared as streaks with R_f_ values in the range 0.28–0.47 and 0.73–1.0 respectively.

Separation of aqueous phase components: Aqueous phase extracts were separated on aluminium backed silica plates using a mobile phase consisting of 0.5% saline/methanol/NH_4_OH (49:49:2 v/v) [34] and silica loosened and counted as described for the lipid phase. Standards consisting of [^3^H]-Choline and [^3^H]-Phosphocholine ([^3^H]PCho) were run and treated as for the cell extracts, betaine and glycerophosphocholine were run and detected using iodine vapour and cytidine diphosphate choline (CDP-choline) was visualised using a UV lamp (UVP, Cambridge UK).

### Enzyme assays

#### Cell lysis

Cells were seeded in 25cm^2^ flasks and treated with metformin as described above ([^3^H-methy] Choline cellular incorporation). After metformin treatment cells were detached with trypsin followed by addition of medium then transferred to microfuge tubes. They were then pelleted by centrifugation at 400g for 5min at 4°C, washed with PBS then suspended in 200μl of homogenisation buffer consisting of 20mM Tris-HCl (pH 7.5) 0.25mM sucrose, 0.5mM dithiothreitol, 1mM aminohexanoic acid and 1mM phenylmethanesulfonyl fluoride. The cells were then lysed by drawing up and down a 25guage needle 10 times. For the CK assay debris was removed by centrifugation at 20,000g for 10min.

#### Choline kinase (CK) assay

To 100μl of CK assay buffer [35] consisting of 40mM Tris-HCl (pH 7.5), 2mM ATP, 10mM MgCl_2_, 0.25mM choline and [^3^H]methyl-choline chloride (74KBq) was added 20μl of cell lysate. After vortex mixing the mixture was incubated in a water bath at 37°C for 1h. The reaction mixture was then diluted with 0.3ml of water and unreacted [^3^H]choline removed by addition 100μl of the ion-pairing agent tetraphenylboron (TPB) in heptan-4-one (5mg/ml) [36]. [^3^H]phosphocholine formed was determined as described above ‘Determination of Phosphorylated [^3^H-methyl] Choline by precipitation’.

#### CTP:phosphocholine cytidylyltransferase (CCT) assay

Cells were treated and lysed as described. A 50μl sample of complete lysate was reserved and the remainder centrifuged at 20,000g for 15min at 4°C. The supernatant was discarded and the pellet (membrane fraction) was suspended in 100μl of homogenisation buffer. CCT activity was assayed in both the complete lysate (total activity) and the membrane fraction by addition of 20μl to 100μl of CCT reaction buffer consisting of 65mM Tris-Hcl pH7.5, 3mM CTP, 10mM MgCL_2_, 2mM EDTA, 45mM NaCl, 0.25mM phosphocholine and [^3^H] phosphocholine (37KBq) [37]. After vortex mixing the mixture was incubated in a water bath at 37°C for 1h. The reaction was then stopped by heating to 100°C for 2min. The product, [^3^H] CDP-choline, was separated from [^3^H]-phosphocholine using TLC as described above for aqueous phase components. Non-radioactive CDP-choline was run on the outer lanes of the TLC plate and visualised using UV to guide removal of the silica from the enzyme assay samples. These were scraped into scintillation vials and [^3^H]CDP-choline determined using scintillation counting.

#### Phosphatidylcholine specific Phospholipase C activity (PtdCho PLC)

PtdCho-PLC activity was determined using a kit (Red phosphatidylcholine-specific phospholipase C assay kit, Amplex, Invitrogen Ltd Paisley UK) following the manufacturer’s instructions.

#### Protein assay

Protein determination was carried out on all lysate preparations for normalisation of the enzyme assays using the bicinchoninic assay (Sigma-Aldrich, UK.

### Cell cycle distribution

Cells were seeded in 25cm2 flasks and incubated at 37°C for 24h then treated with 4 mM metformin for 24, 48 or 72h then harvested by treatment trypsin and after addition of medium transferred to 1.5ml microfuge tubes. They were then centrifuged at 400g for 5min and the supernatant discarded. After two PBS washes they were re-suspended in 0.3ml PBS followed by fixation with 70% ice-cold ethanol during vortexing. The fixed cells were kept at -20°C prior to flow cytometry analysis. For the cell cycle analysis, fixed cells were adjusted to 5x10^5^ cells/ml and washed 2 times with PBS supplemented with 1% albumin. Then the cells were centrifuged at 1000g for 5mins and resuspended in 1ml of staining buffer containing 50ug/ml propidium iodoide, 50ug/ml ribonuclease A, 0.1% v/v triton-x-100 in PBS and incubated for 15 min at room temperature. The stained nuclei were kept at 4°C and protected from light. Flow cytometry was performed using 488 nm laser light on a FACSCalibur flow cytometer (Becton Dickinson) and CELLQuest software (Becton Dickinson) equipped for fluorescence detection, forward, 90° angle light scatter and doublet discrimination. The data analysis was done with Flowjo cell cycling software.

### Statistics

Statistical differences between means were determined using the student’s t test. Tracer incorporation experiments were carried out at least three times with duplicate samples. Enzyme assay experiments were carried out 2 or more times with triplicate or more samples.

## Results

TLC was carried out to identify the [^3^H]choline-containing metabolites and lipids in the aqueous and lipid cell fractions of cells after incubation with [^3^H-methyl]choline followed by a 1h chase. The two lipid standards, phosphatidylcholine and lyso-phosphatidylcholine appeared as streaks with Rf values between 0.28–0.47 and 0.73–1.0 respectively. In lipid samples from control and metformin-treated cells all the [^3^H] appeared between 0.7 and 0.87 indicating that none was present in lyso-PtdCho.

The standards for the aqueous phase Rf values were: Choline 0–0.1; PCho 0.24–0.34; CDP-choline 0.53–0.59 GPC 0.47–0.57; betaine 0.53–0.69. All activity in the aqueous phase extracts of both control and metformin-treated cells appeared at or below an Rf of 0.34 indicating that only [^3^H]Choline and [^3^H]PCho were present in the aqueous phase extracts. Incubation medium from the 60min chase incubations were also subjected to TLC and phosphate precipitation. For all cell lines [^3^H] in medium from the efflux consisted of [^3^H]Choline and [^3^H]PCho.

**Fig 1:**
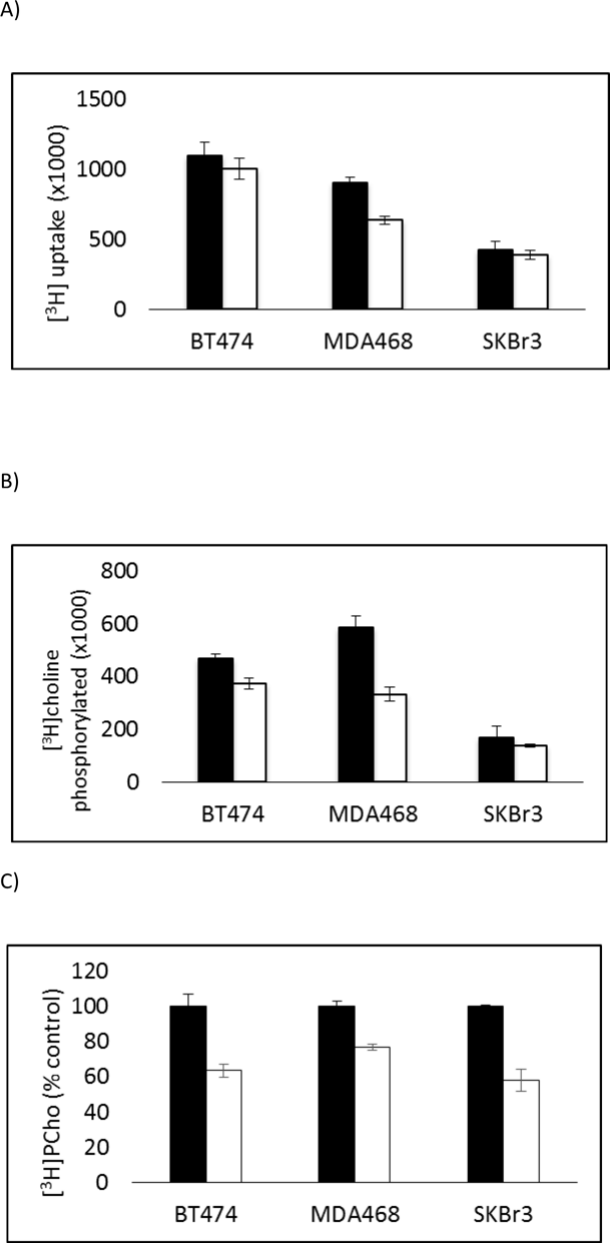
Total [^3^H-methy]choline uptake (A), cellular phosphorylated [^3^H-methy]choline (B) in untreated (solid) and metformin-treated (white) breast tumour cells incubated with [^3^H-methyl]choline for 15min (Units: Radioactive counts normalised to protein content and expressed relative to untreated cells). Choline kinase activity in lysates of untreated (black) and metformin-treated (white) breast tumour cells (C) (Units: [^3^H]PCho formed in 1h normalised to protein content and expressed relative to untreated cells).

The total [^3^H]choline uptake, shown in Fig 1A, by metformin-treated cells was significantly decreased (t = 5.26, p<0.001) in MDA-MB-468 cells but not by either BT474 or SKBr_3_ cells. Cellular [^3^H]choline phosphorylated, shown in Fig 1B, was significantly decreased by all three cell lines after 72h treatment with metformin (BT474 t = 3.57, p<0.01; MDA-MB-468 t = 4.93, p<0.01; SKBr3 t = 2.6.t<0.05). Choline kinase activity was determined in each cell line, shown in Fig 1C, and found to be significantly decreased after treatment with metformin (BT474 t = 4.82, p<0.01; MBA-MB-468 t = 6.45, p<0.001; SKBr3 t = 6.54, p<0.001).

**Fig 2:**
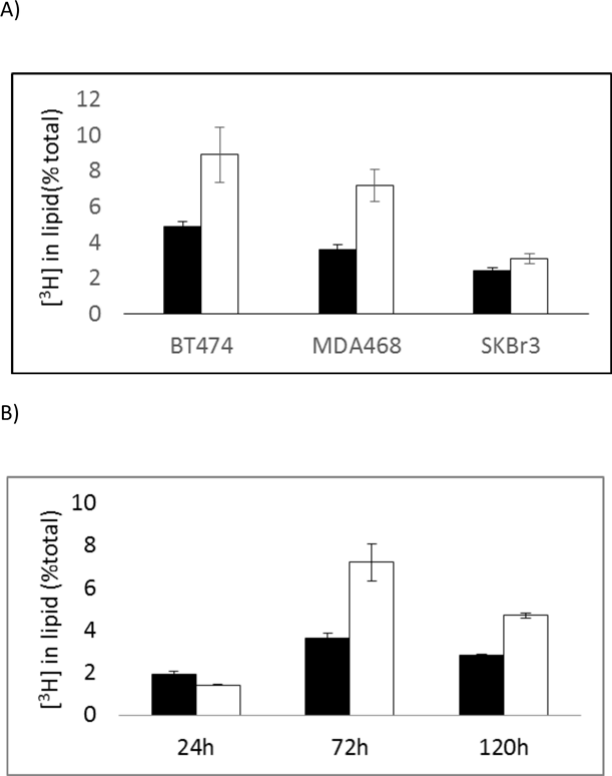
Proportion of [3H-methyl]choline accumulated in the lipid fraction by untreated (black) and metformin-treated (white) breast cancer cells during a 15minute incubation with [^3^H-methyl]choline followed by a 1h chase (medium replaced with non-radioactive medium) expressed as a percentage of total cell [^3^H-methyl]choline uptake. (A) BT474, MDA-MB-468 and SKBr3 cells treated with Metformin for 72 h and (B) MDA-MB-468 cells treated with metformin for 24, 72 and 120h.

Incorporation of [^3^H]choline into the lipid fraction was measured after a 15min pulse with [^3^H]choline in medium and a 1h incubation in non-radioactive medium. Fig 2A shows the incorporation of [^3^H]choline by the lipid extract fraction of BT474 (t = 4.18, p<0.01), MDA-MB-468 (3.42, p<0.01) and SKBr3 (t = 3.2, p<0.05) cells is increased after 72h treatment with metformin compared with untreated cells. Similarly [^3^H]choline incorporation was increased in metformin-treated cells compared with untreated cells at the end of the 15min pulse period with no chase (results not shown). Fig 2B shows that the longer incubation period of MDA-MB-468 cells for 5days with metformin also increased [^3^H]-choline accumulation by lipids (t = 12, p<0.001). Results are shown expressed as [^3^H-methyl]choline uptake by lipid as a percentage of total cell uptake. Similar trends were seen when the results were expressed as [^3^H]-methyl]choline uptake normalised to mg of protein (S1 Fig).

**Fig 3:**
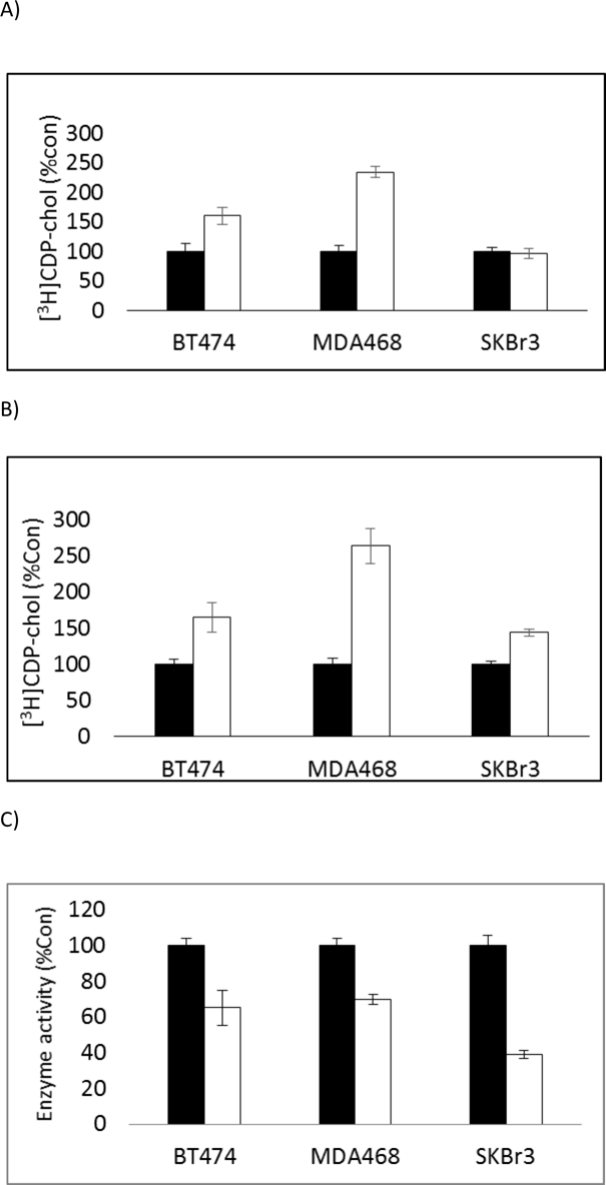
Total (A) and membrane (B) CTP:PCho cytidylyltransferase (CCT) and PtdCho-specific phospholipase C (C) activity of untreated (black) and metformin-treated (white) breast tumour cells. (units: CCT- [^3^H]CDP-choline (CDP-chol) formed after 1h incubation with [^3^H]PCho normalised to protein content and expressed relative to untreated cells; PLC–enzyme units normalised to protein content and expressed relative to untreated cells).

The higher rate of accumulation of [^3^H]choline into lipid in metformin-treated cells may be due to decreased rate of degradation or increased rate of synthesis of PtdCho or both. The formation of CDP-choline catalysed by CCT is the rate limiting step for PtdCho formation. Total CCT activity was increased in BT474 (t = 3.2, p<0.05) and MDA468 cells (t = 9.6, p<0.001) and membrane-associated CCT activity was increased in cell lines treated with metformin (BT474 (t = 5.6, p<0.02), MDA-MB-468 (t = 8, p<0.001), SKBr3 (t = 6.55, p<0.01)). The activity of the catabolic enzyme PtdCho-phospholipase C was significantly decreased (BT474 t = 3.22, p<0.05; MDA-MB-468 6.16, p<0.01; SKBr_3_ t = 9.5, p<0.001) by treatment with metformin (results shown in Fig 3) for 72h.

CCT and PtdCho-PLC activity were also determined after 24h treatment of MDA-MB-468 cells with metformin. PtdCho-PLC activity was significantly (t = 15, p<0.001) decreased by a 24 h treatment with metformin (66 ± 3) compared with control cells (100 ± 1.3) but in contrast to the 72h treatment with metformin membrane CCT activity was not significantly (t = 0.8, not significant) changed (control: 100 ± 15; metformin treated for 24h: 108 ± 23).

The formation of PtdCho from CDP-choline requires diacylglycerol (DAG) which can be formed by several routes including the breakdown of PtdCho and other phospholipids by PLC, de novo synthesis from dihydroxyacetone phosphate and release from triacylglycerol by triglyceride lipase.

**Fig 4:**
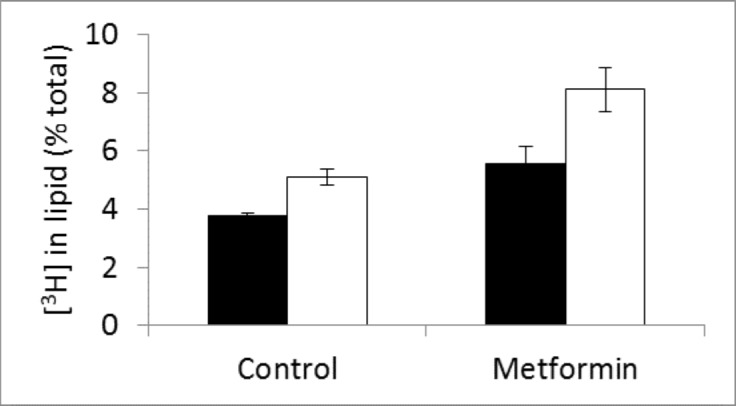
Proportion of [^3^H-methyl]choline accumulated in the lipid fraction by untreated (control) and metformin-treated MDA-MB-468 breast cancer cells incubated for 4h with the PtdCho-PLC specific inhibitor D609 expressed as a percentage of total cell uptake (A).

The lower level of PtdCho-PLC activity in the metformin-treated cells, compared with control cells, suggests that PtdCho-specific PLC activity isn’t a source of increased DAG in metformin-treated cells. Further, treatment of MDA-MB-468 cells with the phosphatidylcholine-phospholipase C inhibitor D609 (100μM) [27] actually resulted in more [^3^H]choline in the lipid fraction of the metformin-treated cells (t = 3.2, p<0.01) (results shown in Fig 4) suggesting that PtdCho-PLC activity breaks down the newly formed PtdCho.

**Fig 5:**
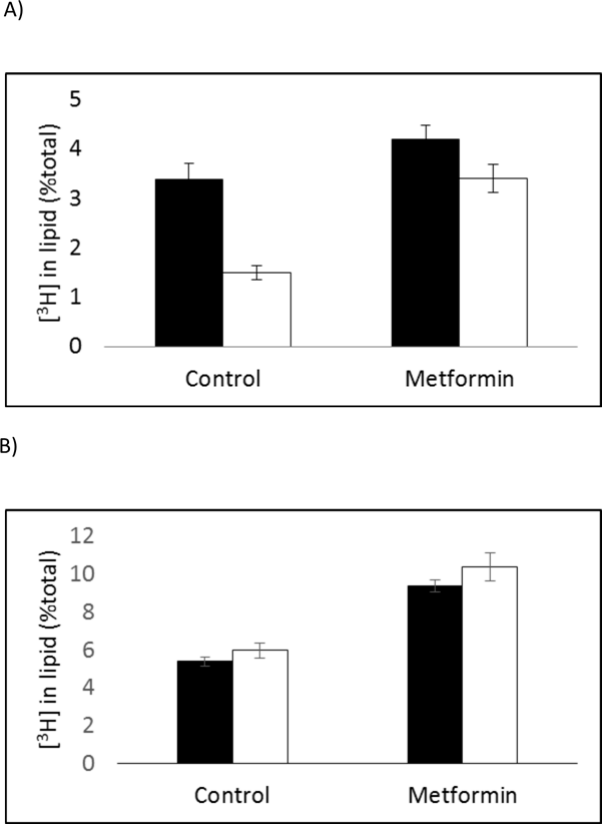
Proportion of [3H-methyl]choline accumulated in the lipid fraction by untreated (control) and metformin-treated MDA-MB-468 breast cancer cells incubated for 4h with the ACC inhibitor TOFA (A) or incubated for 4h with the lipase inhibitor agliastatin (B).

The effect of inhibition of de-novo synthesis of DAG was determined by treatment of control and metformin-treated MDA-MB-468 cells with the acetyl-CoA carboxylase inhibitor, TOFA (20μM) [29] for 4h and choline incorporation into the lipid fraction measured. Lipid [^3^H]choline incorporation (Fig 5A) was found to be decreased by 70% by control cells (t = 5.3, p<0.01) and about 30% by metformin-treated cells (t = 2.85, p<0.05) incubated with TOFA (40μM). This corresponds with the decreased fatty acid synthesis from [14C]glucose in metformin-treated cells. Triacylglycerol breakdown produces DAG but treatment of both control and metformin-treated MDA-MB-468 cells with the inhibitor agliastatin (Fig 5B) did not reduce the lipid [^3^H]choline incorporation suggesting that triacylglycerol breakdown is not an important source of DAG for PtdCho synthesis.

**Fig 6:**
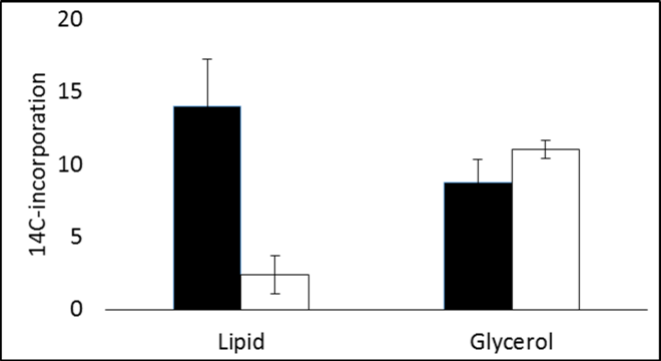
Incorporation of [^14^C] radioactivity into lipid and glycerol by control and metformin-treated cells incubated with [^14^C-U]glucose for 2h after saponification of cell lipid fraction (units [14C]incorporation/mg protein (x1000)).

The proportion of [^14^C] that was accumulated in the lipid fraction of metformin-treated MDA-MB-468 cells (5.4 ± 1.4%) was significantly less (t = 6.1, p<0.001) than that by untreated cells (11 ± 1.7%) after incubation with [^14^C(U)]glucose for 2h. To discriminate the amount incorporated into glycerol and into fatty acids the lipid fraction was saponified to release the glycerol from fatty acids and the fractions separated. The results are shown in Fig 6. The amount of activity associated with glycerol was significantly increased (t = 3.2, p<0.05) by metformin treatment compared with untreated cells but the amount in the lipid extract (fatty acids) was found to be greatly decreased (t = 8.2, p<0.001).

**Fig 7:**
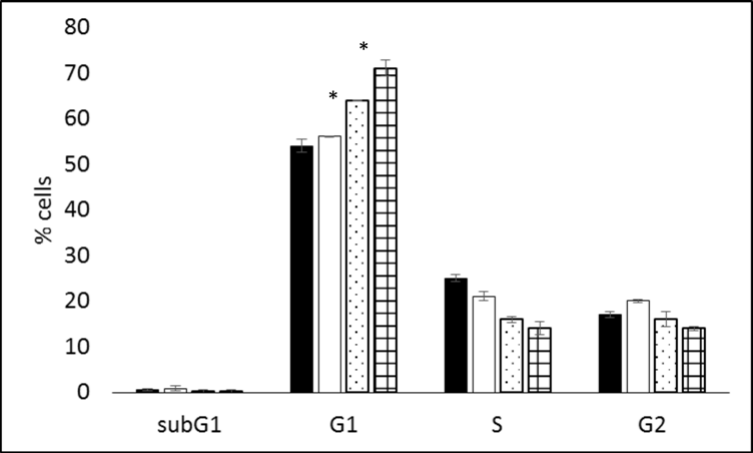
Cell cycle distribution in MDA-MB-468 cells treated with metformin for 24h (white), 48h (speckle) and 72h (squares).

PtdCho synthesis may vary through the cell cycle so control and metformin-treated MDA-MB-468 cells were subject to cell cycle analysis. Treatment with metformin for 48 (p = 10.8, t<0.001) and 72h (t = 12.7, p<0.001) but not 24h resulted in a significant increase in cells in G_0_/G_1_ with a concomitant decrease in cells in S-phase after 48 (t = 13.7, p<0.001) and 72h (t = 13, p<0.001) metformin treatments compared with controls (Fig 7).

## Discussion

**Fig 8:**
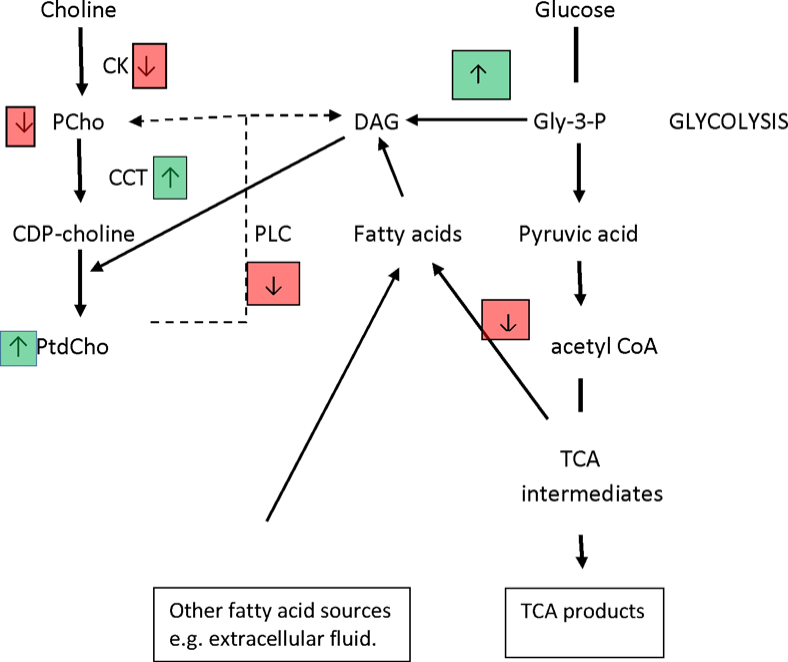
Alterations in phosphatidylcholine metabolism and conversion of glucose to fatty acid and glycerol associated with metformin treatment. (Abbreviations: PtdCho, phosphatidylcholine; PCho phosphocholine; CDP-choline, cytidine tri-phosphate–choline; CK, choline kinase; CCT, CTP:phosphocholine cytidylyltransferase; DAG diacylglycerol; PLC phospholipase C; TCA, tricarboxylic acid; Gly-3-P, glyceraldehyde-3-phosphate.

This study has demonstrated that treatment of breast cancer cells with metformin is associated with modifications in the activity of the enzymes choline kinase, CCT and PtdCho-PLC with corresponding changes in the incorporation of choline into Pcho and PtdCho. Metformin treatment was also found to be associated with modulated fatty acid and glycerol formation from glucose. These metformin associated changes are summarised in Fig 8.

Metformin is currently undergoing clinical trials [38] for the treatment of cancer, however the mechanism of metformin’s tumour growth inhibition is poorly understood [38]. Malignant transformation has been shown to be associated with increased cellular PCho [4] content which is attributed to increased choline kinase activity in breast cancer [6,7]. Previous studies have shown that choline kinase [39] and phospholipase C activity [40] are important in cell survival and their inhibition results in decreased cancer cell proliferation. Decreased choline kinase and phospholipase C activities associated with metformin treatment, demonstrated in this study, may be amongst the mechanisms of tumour cell growth inhibition induced by metformin.

In agreement with previous studies [38] including studies of breast cancer cells [41], treatment of cells with metformin was found to induce G_1_ arrest in tumour cells which was apparent after 48 and 72h of treatment. Previous studies have demonstrated that PCho content correlates with S-phase fraction [36,42]. PCho content was lower in cells treated with metformin for 72h corresponding with the lower S-phase fraction in these cells. However inhibition of choline kinase and cell cycle distribution by metformin may be independent events.

Saponification of the lipid fraction of cells incubated with [^14^C]glucose facilitated determination of the fraction of glucose-derived [^14^C] activity associated with fatty acids and glycerol. The latter, which is derived from glyceraldehyde-3-phosphate during glycolysis, was increased corresponding with increased glucose utilisation by tumour cells treated with metformin [21, 43]. Increased glucose utilisation in breast cancer stem cells treated with metformin has been shown to be accompanied by increased lactic acid production [43] but decreased ATP synthesis. Zakikhani et al [44] have also shown that metformin treatment of MCF7 breast cancer cells increased glycolysis and concomitantly lactate production but decreased the concentration of tricarboxylic acid (TCA) cycle intermediates including citrate (TCA) which is a major source of fatty acids as well as of ATP. Here the glucose derived fatty acid fraction from lipids derived from [^14^C]glucose was found to be greatly decreased in cells treated with metformin.

Cancer cells exhibit enhanced fatty acid synthesis which is essential for the production of complex lipids e.g. phospholipids for cell signalling and growth [45]. PtdCho-PLC degradation of PtdCho is a source of DAG [46]. Abalsamo et al [27] have shown that PtdCho PLC, but not PLD is increased in breast cancer cells compared with non-tumour cells and that inhibition of PLC by D609 induces cell cycle arrest. CCT is controlled by intracellular DAG level [47, 48]. Here PLC activity was found to be decreased by treatment of breast cancer cells with metformin however the increase in lipid produced from de novo synthesised glycerol may increase DAG content so increasing CCT activity. Alternatively, increased membrane CCT activity, which is evident at 72h but not 24h, may be a compensatory effect of decreased accumulation of choline by PtdCho observed at 24h but which then results in a higher rate of accumulation at later time points. This mechanism has previously been described for liver cells treated with the AMPK activator AICAR [49]. Houweling et al [49] observed that membrane CCT activity was increased in hepatocytes treated with AICAR though they did not report increased accumulation of choline by PtdCho. However as PtdCho accumulation is a net effect of anabolism and catabolism its accumulation may not always be evident in response to increased CCT activity.

Although de novo fatty acid synthesis is decreased by treatment with metformin, [^3^H]choline accumulation into PtdCho is increased indicating that alternative sources of fatty acids must be utilised by breast cancer cells treated with metformin. Possible sources include those derived from the extracellular fluid and ones synthesised from acetate. Breast cancer cells have been shown to utilise fatty acids present in serum in the incubation medium [50]. Acetyl-CoA for de novo synthesis of fatty acids can be formed by the acetyl CoA synthase (ACSS) catalysed ligation of acetate with CoA [50]. A recent study has shown that exposure of breast cancer cells to hypoxia which, in common with metformin induces metabolic modifications by increased AMPK activity, exhibited enhanced fatty acid synthesis from acetate by increasing the expression of an isoform of ACSS. Schug el al [51] also demonstrated that hypoxia decreased the conversion of glucose to fatty acids whilst maintaining glycerol-3-phosphate production for lipid synthesis.

Increased lipid incorporation of [^3^H]choline incorporation was detected in each cell line treated with metformin, but the magnitude of the effect was greater in MDA-MB-468 and BT474 cells than in SKBr3 cells. Studies e.g. [52] have shown that ER status can influence fatty acid synthesis whilst metformin-treatment can be beneficial in patients refractory to anti-HER treatment [53]. Although biologically the cell lines are distinct in their receptor expression (MDA-MB-468 cells are triple negative cells (don’t express the oestrogen (ER+) or progesterone (PR+) receptors or HER-2) but over-express EGFR, BT474 and SKBr3 cells over-express HER-2 but not EGFR. BT474 cells are ER+ and PR+ and SKBr3 are ER- and PR -) no single receptor differences between the cell lines could account for the relatively lower effect of metformin on lipid incorporation of [^3^H]choline incorporation.

Several studies have shown that anticancer treatment response [36, 54–55] is accompanied by reductions in tumour PCho content which is considered a potential marker of treatment response. Decreased PCho in metformin-treated cells compared with untreated cells suggests that monitoring PCho levels or choline utilisation using NMR spectroscopy or [^11^C]choline/[^18^F] Fluoro-choline positron emission tomography (PET) respectively [56] may be useful imaging methods for detecting response of tumours treated with metformin.

In conclusion we have shown that treatment of breast cancer cells with metformin increases accumulation of choline by phospholipid, increases CCT activity and decreases choline kinase and PLC activity. We have also shown that the glycerol fraction derived from glucose is increased by breast cancer cells treated with metformin which may contribute to increased levels of DAG for PtdCho formation. We have also confirmed that the formation of glucose-derived fatty acids is decreased in metformin treated cells. Techniques that measure phosphatidylcholine metabolism including [^11^C]choline-PET may be useful in measuring cancer cell response to metformin.

## Supporting Information

S1 Fig Lipid uptake of [3H]choline expressed as relative to total protein (DPM/mg protein). (DOCX)
