# Supplementary material for: Metformin Decouples Phospholipid Metabolism in Breast Cancer Cells
Source: PLoS One. 2016 Mar 9;11(3):e0151179. doi: 10.1371/journal.pone.0151179 (PMC4784930; doi:10.1371/journal.pone.0151179)
Supplement: S1 Fig — (DOCX) [file pone.0151179.s001.docx]

Data for lipid uptake of [3H]choline expressed as relative to total protein (DPM/mg protein)

Figure
